# Health Technology Assessment for the Prevention of Peri-Operative Hypothermia: Evaluation of the Correct Use of Forced-Air Warming Systems in an Italian Hospital

**DOI:** 10.3390/ijerph20010133

**Published:** 2022-12-22

**Authors:** Giulia Zucconi, Anna Maria Marchello, Camilla Demarco, Elisabetta Fortina, Ljdia Milano

**Affiliations:** 1 ASST Lodi, 26900 Lodi, Italy; 2 ASST Ovest Milanese, 20025 Legnano, Italy; 3 Lundbeck Italia Spa, 56021 Cascina, Italy; 4 ASL Novara, 28100 Novara, Italy; 5 Hospital Consulting Spa, 50012 Bagno a Ripoli, Italy

**Keywords:** hypothermia, normothermia, inadverted perioperative hypothermia, forced-air warming, active warming, temperature management, surgical patient

## Abstract

This study investigates the implications of using a system for the maintenance of normothermia in the treatment of patients undergoing surgery, determining whether the FAW (Forced-Air Warming) systems are more effective and efficient than the non-application of appropriate protocols (No Technology). We conducted Health Technology Assessment (HTA) analysis, using both real-world data and the data derived from literature, assuming the point of view of a medium-large hospital. The literature demonstrated that Inadvertent Perioperative Hypothermia (IPH) determines adverse events, such as surgical site infection (FAW: 3% vs. No Technology: 12%), cardiac events (FAW: 3.5% vs. No Technology: 7.6%) or the need for blood transfusions (FAW: 6.2% vs. No Technology: 7.4%). The correct use of FAW allows a medium saving of 16% per patient to be achieved, compared to the non-use of devices. The Cost Effectiveness Value (CEV) is lower in the hypothesis of FAW: it enables a higher efficacy level with a contextual optimization of patients’ path costs. The social cost is reduced by around 30% and the overall hospital days are reduced by between 15% and 26%. The qualitative analyses confirmed the results. In conclusion, the evidence-based information underlines the advantages of the proper use of FAW systems in the prevention of accidental peri-operative hypothermia for patients undergoing surgery.

## 1. Introduction

Inadvertent Perioperative Hypothermia (IPH) is a known complication that can occur in patients undergoing surgery and it is defined by a Central Body Temperature (CT) below 36 °C [1,2], which occurs as a result of the suppression of the central temperature regulation mechanisms caused by anesthesia and a prolonged exposure of large areas of skin to cold temperatures in operating theatres [3,4].

It is therefore an anesthesia-associated event that alters the body’s thermoregulatory capacity, impairing the peripheral vasoconstriction and thermogenesis mechanisms. In both general and local anesthesia, the CT trend is a triphasic curve: it has a faster downward slope in the first phase (redistribution phase), a slower downward slope in the second phase, and finally a stabilization phase. Under general anesthesia, for example, within 30 min of induction, there is a decrease of 1 °C in the CT, and three to four hours after induction, a temperature loss of 2.8 ± 0.5 °C can be achieved [5].

IPH is defined as a frequent event and estimates of the prevalence of hypothermia are in a rather wide range, between 50% and 90% [6,7].

It has been shown that inadvertent hypothermia, even in non-serious cases, causes significant alterations to the cardiovascular system, the endocrine and metabolic systems, and causes the blood to crash [5]. IPH has been associated with clinically relevant adverse consequences, such as surgical site infection, delayed wound healing, increased bleeding, cerebrovascular ischemic events and, through the inhibition of coagulation, hemorrhagic events [1,2].

In trauma patients, IPH results in an increased incidence of mortality [8]. Surgical wound infections represent one of the main causes of postoperative morbidity and prolonged hospitalization leading to a higher cost of hospital care. In the period between 2016 and 2017, the European Centre for Infectious Diseases developed two studies on care-related infections and antibiotic use [9]. According to the data reported by the corresponding Italian PPS2 Report 2016/2017 [10], in Italy, the probability of contracting infections during a hospital stay is 6%, with 530 thousand cases each year.

These complications can be significantly reduced by resorting to good clinical practices to prevent accidental peri-operative hypothermia [11]. In fact, in most cases, IPH is proactively preventable through CT monitoring and patient warming in all peri-operative phases [1,4,8,12]. For this reason, several guidelines [1,2,13] recommend central temperature monitoring in both general anesthesia and loco-regional anesthesia interventions for adults, with a duration of more than 30 min. In Italy, the SIAARTI (Società Italiana Anestesia, Analgesia, Rianimazione, Terapia Intensiva) Good Clinical Practices [2,14], as well as the NICE (National Institute for Health and Care Excellence) [1] for the United Kingdom, recommend that the patient should be discharged from the operating theatre with a core temperature of at least 36 °C.

Systems that use the principle of heat transfer during anesthesia have been available for several decades, and numerous studies have demonstrated their effectiveness in preventing peri-operative hypothermia. Nevertheless, according to a prevalence study published in France at the end of 2019, incidences of hypothermia on admission to the recovery room remains very high in percentage terms [15]. Alfonsi et al. [15] found that among 893 subjects (mean age 66.9 years), the prevalence of hypothermia on admission to the recovery room was 53.5%, despite the fact that at least one heating system had been used for 90.4% of the patients. According to Alfonsi [15], only a combination of preheating and intraoperative heating prevented a CT < 36 °C (OR = 0.48 [CI95%: 0.24–0.96]).

At present, several passive or active warming methods for the peri-operative period are available. Passive warming techniques include the use of cotton blankets and other covers, such as surgical drapes, which act to minimize body heat loss through increased insulation [16]. These passive techniques, used until about 1990, have been replaced by active techniques that use air, water, or electricity to enhance the warming process. In addition to reducing heat loss, active skin warming techniques provide heat to the patient through a variety of means, including air and water systems. According to the Cochrane systematic review of 67 Randomized Clinical Trials (RCT) [17], which analyzed different active temperature maintenance systems, forced-air warming (FAW) appears to have a beneficial effect in terms of a lower rate of surgical site infections and complications compared with no active warming system. Forced-air warming also has a beneficial effect on major cardiovascular complications and improves patient comfort. In addition, the effect on blood loss is also statistically significant, although this difference does not always translate into a substantial reduction in transfusions. Evidence for other active body surface warming solutions are scarce in the literature; however, they indicate a beneficial effect on shivering and tremors similar to electrical or resistive warming systems.

Some evidence suggests that extending systemic warming to the pre-operative period may be more beneficial than limiting it to only during surgery. A very recent study, in 2020 [18], has shown that a post-operative warming process has a positive impact on multiple post-operative outcomes. This is a groundbreaking result, as previous studies have focused on pre- and intra-operative warming [19,20]. Although the time interval between patients’ admission to the Intensive Care Unit (ICU) and the initiation of FAW was significantly longer compared to the use of passive solutions (blanket), patients who were exposed to a FAW system achieved normothermia earlier than those using a traditional blanket. Both methods were equally effective in warming patients up to 2 h, but after 2 h, the FAW system was more effective and only 15 h later the groups were equal in terms of mean temperature. Similarly, a previous study [21] dealing with laparoscopic surgery found the effectiveness of FAW is the same as the passive heating method after the start of anesthesia and during the first 30 min of surgery; however, after 90 min, the FAW system proved to be more effective. Brauer et al. [22] report that the use of FAW after cardiac surgery increases the rate of warming by two to three times compared to the conventional blanket. According to Madrid et al. [17], the difficulty of associating clinically relevant benefits other than temperature with active body surface warming systems can be explained by the fact that many studies have applied concomitant procedures that are routinely in place as co-interventions to prevent hypothermia as passive or active warming systems based on other physiological mechanisms (e.g., irrigation fluid or gas warming).

Based on the above considerations, no consensus exists in the Italian setting concerning the use of FAW in clinical practice, thus requiring an in-depth analysis of its potential and incremental benefits with respect to the standard of care. According to this, the present paper aims to define the impacts related to FAW in terms of clinical and economic efficiency (for patients and hospitals), thus also evaluating the potential organizational advantages for hospitals caring for such patients. In particular, the main objective is to define if there is a real benefit to the introduction of a system for the maintenance of normothermia in patients undergoing surgery with a surgical time greater than 30 min, as well as determining whether the use of convective warm air systems (FAW) is more effective and efficient, in terms of a reduction in the occurrence of adverse events, length of stay (LOS) and costs, than the non-application of appropriate protocols (not using any device—“No Technology”), in the hospital setting.

The achievement of such objective will enable us to adequately answer the following policy question: “Does the proper use of FAW prevent accidental perioperative hypothermia in patients undergoing surgery, with a consequent achievement of significant benefits, assuming both the hospital and the patients’ point of view?”

## 2. Methodology

Health Technology Assessment (HTA) analysis was conducted, assuming the point of view of a medium-large hospital located in Northern Italy, in order to compare the innovative technology of the FAW with the comparator “no technology” and taking into consideration three sample specialties: General Surgery, Orthopedics and Neurosurgery. However, the analysis can be extended to all surgical specialties.

The HTA assessment examined all of the relevant dimensions of the EUnetHTA Core Model [23], which is the most acknowledged approach when evaluating innovative technologies in all European Countries. These include: (i) health problems and the current use of technology, (ii) description and technical characteristics, (iii) safety, (iv) clinical effectiveness, (v) cost and economic evaluation, (vi) ethical analysis, (vii) organizational aspects, (viii) social aspects, (ix) legal aspects.

These dimensions were investigated through a combination of different data taken from scientific evidence found in the literature (health problem and current use of technology, description and technical characteristics, safety, efficacy), information collected through the direct involvement of professionals working in the perioperative process (qualitative analysis: safety, effectiveness, ethical analysis, qualitative organizational aspects, social aspects, legal aspects), and data collected from the reality subject of analysis (quantitative analysis: cost and economic evaluation, social aspect, quantitative organizational aspects).

## 3. Literature Review

The first fundamental step in each round of HTA analysis is the evidence assessment, or review of the extant literature, taking into account the evidence available on the topic and valuating their robustness.

For this reason, in order to obtain an adequate documentary basis to proceed with the examination of the dimensions of HTA, a systematic review of the available literature concerning the prevention of inadvertent peri-operative hypothermia through the proper use of forced-air warming systems was carried out, according to the following P.I.C.O. method:

*P (Population):* adult patients (age ≥18 years) undergoing surgery lasting longer than 30 min, either under general or loco-regional anesthesia.

*I (Intervention):* the appropriate use of Forced Air Warming (FAW) technology.

*C (Comparator):* the absence of patient warming systems.

*O (Outcome):* the reduction in adverse events associated with accidental peri-operative hypothermia and their consequences in terms of prolonged hospital stay.

From this information, a search strategy containing several keywords (including surgical or anesthetized patient, warm system, temperature drop, temperature management, inadvertent or unintended hypotherm *, normotherm *, forced air, faw, active warming, conductive warming, convective warming, air convection) was designed and was inserted into three databases: PubMed, BioMedCentral and Cochrane Library.

To select the appropriate papers to perform the analysis, the following eligibility criteria were chosen: adult patients (older than 18 years), duration of surgery longer than 30 min, study on the topic of interest, number of participants in the study greater than 100, papers published since 2000, language (Italian or English). To reduce the number of selected studies, the decision was made to further narrow the inclusion criteria, with the additional criteria: disciplines selected in the analysis, studies published since the year 2010, complete studies only (excluding all studies of which only the abstract is present), studies with full text online accessible only.

The validation of the available scientific evidence on the topic was performed through the AMSTAR II scale [24] (for systematic reviews and meta-analysis), the NEW CASTLE OTTAWA SCALE [25] (for cohort study), and the JADAD scale (for RCT study).

Overall, through the analysis of the literature, it was possible to derive data from studies already available and use them to investigate the following dimensions of analysis: health problem and current use of technology, description and technical characteristics, safety, and efficacy.

## 4. Quantitative Analysis

The calculation of the actual and potential target population, as well as investigations of the dimensions of cost and economic evaluation, social aspects, and organizational aspects (quantitative), were conducted through quantitative analysis.

Due to the fact that the scientific evidence have been evaluated as medium-high quality, the safety and efficacy data obtained from the literature were used to carry out the quantitative analysis.

### 4.1. Target Population

Starting from the Italian population, the analysis focused on hospitalizations with surgical Diagnosis Related Groups (DRGs) provided in the year 2019 to adult patients, with surgery time greater than 30 min. The analysis was performed on both nationwide aggregate data and data extracted from the operating room databases of a medium-large hospital located in Northern Italy. In order to properly define User Group in scope, several sources were examined: ISTAT (Istituto Nazionale di Statistica) website [26] for information about population demographic data at national and regional level, SDO (Scheda di Dimissione Ospedaliera) Report 2019 [27] (Ministero della Salute) for health data at national and regional level, hospital internal database for health data at local level.

### 4.2. Cost and Economic Evaluation

To implement the cost and economic evaluation, process analysis, a complete economic-health assessment, and analysis of the direct financial impact on the hospital were carried out.

The process analysis was conducted using the Activity Based Costing (ABC) technique [28], identifying resources used and standardizing the planned surgical pathway of a patient into four phases: (i) pre-operative hospitalization in the acute care unit; (ii) surgery; (iii) post-operative ICU hospitalization; (iv) post-operative hospitalization in the acute care unit. The costs were determined for each phase, using these components:

- Pre- and post-operative hospitalization phase: the average cost of pre-operative hospitalization (average cost of a single day of hospital stay—personnel costs, food, drugs, etc.—multiplied by the average pre-operative hospitalization), average cost of post-operative hospitalization in cases of normothermia and in cases of hypothermia (reduction in the number of post-operative hospitalization days due to the reduction in adverse event occurrence).
- Post-operative ICU hospitalization: the average cost of ICU hospitalization for each day of hospitalization in the ICU.
- Surgery: the average hourly cost of the operating room, the average cost of surgery.
- Devices: the average cost of the FAW devices, the average cost of annual maintenance, the average cost of consumables for the FAW devices.
- Adverse events: the average cost, per patient, for the resolution of adverse events (infections, transfusions, cardiac events).

Finally, the weighted average of the process costs in cases of hypothermia (failure to use adequate temperature maintenance systems) and normothermia (use of FAW systems to maintain body temperature) were calculated, considering the number of surgical interventions performed in a year, in the reality under examination.

To carry out a comprehensive health and economic evaluation, a cost-effectiveness analysis [29] was performed by calculating the ratio between real process costs and effectiveness value found in literature (Cost Effectiveness Value—CEV) and quantifying the incremental cost-effectiveness value (Incremental Cost Effectiveness Ratio—ICER).

Ultimately, a Budget Impact Analysis (BIA) was conducted [30], comparing the baseline scenario (actual data from the hospital considered in which a minimum percentage of patients are treated with FAW devices) with three innovative scenarios (data taken from the literature review where protocols for normothermia are correctly adopted and progressively higher percentages of patients are treated with FAW devices), over a time period of 12 months.

### 4.3. Social Aspects

The social cost analysis exclusively considers the evaluation of patients’ productivity loss due to a prolonged episode of hospitalization because of adverse events triggered by an inappropriate use of the protocol regarding normothermia.

To conduct this analysis, the average length of stay in normothermic (FAW device use) and hypothermic (no device use) conditions was calculated. By valuing the days of hospitalization in the two scenarios at an average daily value of the wage, the differential social cost was obtained.

### 4.4. Organizational Aspects

The quantitative organizational impact dimension is useful for planning the supplementary investments required for the use of new technology, for example, personnel and staff training, additional space, equipment, or software. To examine this dimension, scenarios used to conduct the BIA were taken into consideration, using the difference in terms of inpatient days and calculating the differential between the alternative hypotheses, to define if the FAW introduction would lead to a reduction in hospital bed occupancy.

### 4.5. Qualitative Analysis

The qualitative questionnaires taken from the EUnetHTA Core Model [23] were administered to a multi-disciplinary group of 12 healthcare professionals directly involved in the peri-operative patient pathway. Among these, two were anesthesiologists, two were neurosurgeons, two were cardiac surgeons, two were perfusion technicians, and four were operating room nurses.

Through the analysis of the responses collected from the questionnaires (seven-item Likert scale ranging between −3 and +3 [31]), it was possible to investigate the dimensions of safety, clinical effectiveness, ethical analysis, social aspects, legal aspects, and qualitative organizational aspects.

In the table below (Table 1), the main aspects investigated for each dimension are described:

## 5. Results

### 5.1. Results from Literature Review

The literature research conducted in November 2020 resulted in 650 records, of which 624 were identified through the database search and 26 additional records were identified through other sources as guidelines. After the removal of duplicates, 626 records were screened. Overall, only 125 records were assessed for eligibility. The other 501 records were excluded because they did not meet the chosen eligibility criteria: adult patients, duration of surgery longer than 30 min, study on the topic of interest, number of participants in the study greater than 100, papers published since 2000, Italian or English language. 

To reduce the number of selected studies, the decision was made to further narrow the inclusion criteria, adding the following: specialties selected in the analysis (General Surgery, Orthopedics and Neurosurgery), studies published since the year 2010, complete studies only (excluding all studies of which only the abstract is present), studies with full text online accessible only. Out of 125 records, 120 full-text articles were excluded, according to these criteria.

**Figure 1:**
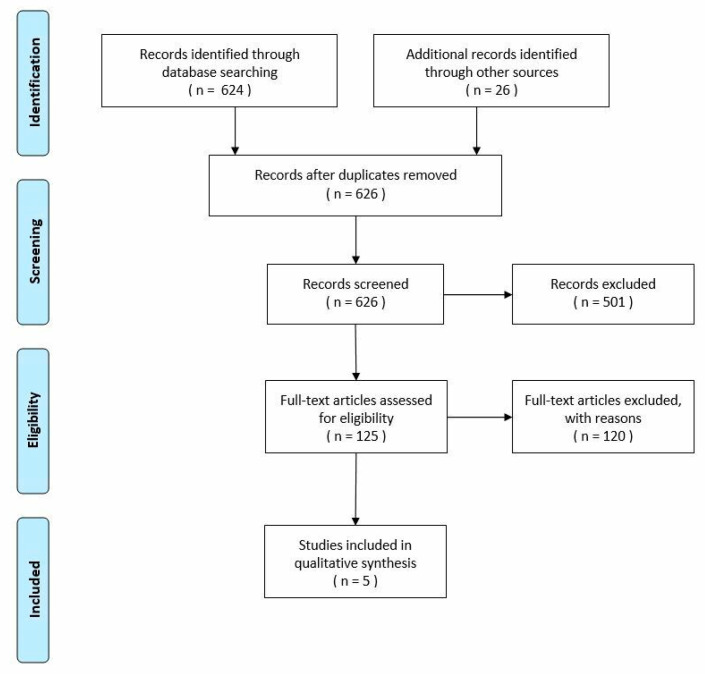
Dimensions and features investigated in the qualitative analysis PRISMA flow chart.

At the end of the process, only five studies met the inclusion criteria. The literature review is presented in the following PRISMA flow chart (Figure 1): 

In January 2021, another recently published study was included in the analysis.

The validation of the scientific evidence performed by two independent valuators revealed a medium-high quality of the studies, as presented in the table below (Table 2):

### 5.2. Safety and Efficacy

The literature review allowed us to find evidence-based information in reference to the dimensions of safety and efficacy indicators (Table 3).

From a safety perspective, IPH, or low IPH, determines clinically relevant adverse events, such as surgical site infection, delay in wound healing, increased bleeding, increased cerebrovascular ischemic events and hemorrhagic events [1,2]. Moreover, in patients with trauma, IPH generates an increased incidence of mortality [8]. These worsening outcomes lead to a prolongation of post-operative hospitalization time and, therefore, of the hospital stay with a consequent increase in costs incurred by the Hospitals.

According to Madrid et al. [17], there is no evidence to suggest that active body surface warming systems pose a significant risk to patients. In addition, the data from the NICE [1] show some adverse events related to the development of hypothermia, which may suggest that the use of specific devices may improve the overall safety indicators.

Focusing on the efficacy indicators of the two compared technologies, Table 3 depicts the parameters derived from the literature review, concerning both the incidence of IPH and the incidence of adverse events in cases of FAW technology use or in cases of no usage of any device. Among the most severe and frequent complications that can be significantly reduced by using right clinical practices to prevent IPH is surgical wound infection [11]. In Italy, the probability of contracting infections during hospitalization is approximately 6%, with 530.000 events occurring every year [10]. In consideration of this high rate, IPH should be avoided throughout the whole peri-operative path in order to reduce the incidence of already frequent adverse events.

## 6. Results from Quantitative Analysis

### 6.1. Cost and Economic Evaluation

Table 4 shows the absorption of economic resources related to the presence or absence of adequate medical devices to prevent IPH.

In particular, the pre-operative hospital stay costs, the surgery costs and the intensive care hospital stay costs are the same because, in the first and in the second cases, the scenarios are identical, while in the third case there is no scientific evidence in the literature that shows a difference in cases of hypothermia or normothermia.

The FAW technology costs are different because the technology is only used in normothermia scenarios, while the cost for adverse events and the post-operative hospital stay costs are higher in cases of hypothermia due to the higher incidence rate of adverse events, according to the literature, that cause an increase in the number of hospitalization days.

Overall, the correct use of FAW technology achieves a saving of 16%, equal to 2131.76 EUR, per patient.

Moreover, cost-effectiveness analysis was carried out, through the definition of the average cost-effectiveness value (CEV), considering the efficacy parameters “surgical wound infections” and “cardiovascular complications”. Both parameters have been translated into positive value and consider, in the first case, the percentage of patients who do not register infections of the surgical wound after surgery and, in the second case, the percentage of patients who do not register cardiovascular complications after surgery.

In both cases, the CEV is lower in the hypothesis of adequate usage of FAW systems, compared to the absence of any devices: the FAW technology is the preferable or “dominant” solution, because it allows to reach a higher efficacy level with a contextual optimization of the patient’s path costs.

To better understand the financial impact of the adoption of FAW technology, budget impact analysis has been conducted (Table 5). It considers a time period of 12 months, an as-is scenario with a low presence of FAW technology and three to-be scenarios with a gradual introduction of FAW technology (usage rates and consumption data are taken from literature—Monzani et al. [39]). The introduction of FAW devices allows a significant reduction in direct costs, varying between −7.61% and −13.20% for the treatment of 8.544 patients undergoing surgery, according to the FAW penetration rate within the Italia clinical practice.

### 6.2. Organizational and Social Quantitative Aspects

The advantages of FAW devices would not be relegated to the economic sphere. Given the fact the FAW would significantly reduce the overall length of stay (weighted average hospital stay in case of hypothermia is equal to 8.45 days; weighted average hospital stay in case of normothermia is equal to 5.85 days), important social and organizational benefits may occur.

From an organizational perspective, in the attempt to quantify the release in the occupation time of a single bed, FAW technology would lead to a release in hospital stay ranging between a minimum of 15% and a maximum of 25% (Table 6), thus also leading to an increase in the overall accessibility to care.

Furthermore, a different length of stay would generate a significant benefit in terms of social costs. Thus, in taking into consideration the patient’s productivity loss related to an episode of prolonged hospitalization of a patient, caused by adverse events generated by inappropriate use of the normothermia procedure, FAW would reduce the social costs by 30.77% (Hypothermia: 612.63 EUR versus Normothermia: 424.50 EUR)

## 7. Results from Qualitative Analysis

Table 7 details the results of the questionnaires administered to the healthcare professionals involved in order to define their perceptions with regard to the routine implementation of FAW in clinical practice.

The data from the qualitative questionnaires confirmed the results of the previous analysis, demonstrating that FAW technologies are advantageous in all of the investigated dimensions. Regarding the safety and effectiveness dimensions, in the case of not using temperature maintenance systems, no adverse events will occur due to technology, but certainly the temperature control and maintenance are not guaranteed (Safety = FAW:1.38 vs. No Technology:−0.46; Effectiveness = FAW:1.73 vs. No Technology:−1.20). Concerning the organizational impact, negative scores were assigned for FAW technology only in cases of a request for additional staff and the impact of electricity consumption. In the long term, compared to a shorter time period, the ease of preoperative set-up and the ease of maintenance of the devices are more favorable.

## 8. Discussions

Intraoperative active warming reduces the incidence of more severe complications, particularly infectious and cardiovascular events, as well as also reducing the number of peri-operative blood transfusions, mitigating the most common adverse events reported in hypothermic patients, such as prolonged recovery time from core temperature and waking up in the recovery room, in addition to the psychological stress of shivering [17,36,40].

Despite data supporting active warming during surgery and the availability of effective warming systems, the prevalence of peri-operative hypothermia remains highly variable between healthcare facilities, ranging between 4% and more than 70% [41,42]. Furthermore, several studies have reported a high rate of hypothermic patients (TC < 36 °C) being admitted to the recovery room despite the use of heating systems [7,42,43].

Although these issues have also been emphasized in national and regional recommendations [1,2,44,45,46,47], in clinical practice, these behaviors are not always correctly applied, demonstrating a real-world evidence gap.

This finding unfortunately confirms the investigations at both international and national levels. According to the results of a survey published in 2017 by SIAARTI [14], in 80% of Italian hospitals there is no specific protocol shared with surgeries and Emergency Departments for the prevention of hypothermia or for the peri-operative monitoring of patients. In Europe, the situation could be considered similar: the results of a survey conducted in 316 European hospitals (TEMMP—Thermoregulation in Europe Monitoring and Managing Patient Temperature—study [48] on 8.083 procedures performed) indicate that the percentage of surgeries during which the patient’s temperature is monitored is 19.4% and the percentage of patients who are warmed is 38.5%. The survey also showed the differential between general and local anesthesia practices: 43% of patients are warmed under general anesthesia and 28% under local anesthesia. The most frequently used heating method is forced-air warming (FAW).

It is important to underline that a higher level of safety for the patients could be achieved with a general economic saving, demonstrating the fact that the introduction of medical devices able to solve organizational process and improve the delivery of services process could be beneficial for hospitals.

Therefore, the “per process” vision should reinforce the opportunity to save money, even with the higher costs of the medical devices and pharmacy items.

Accordingly, it is worth emphasizing that only the appropriate use of the technology, through strict adherence to protocols, can obtain the advantages described, both from a clinical and economic point of view.

The limits of the present study are related to the fact that the original data are based on a sample of patients from a single hospital and the professionals that have collaborated by answering the survey work in the same structure. Thus, it would be useful to integrate the analysis with answers of experts from other hospitals and data derived on more than one structure.

In addition, the administration of questionnaires about quality of life to the patients throughout the hospital stay could integrate the study, proving the real benefits of the technology on the patient experience, and be relevant for further quality of life studies in other aspects of medicine, in order to enable clinicians to align their treatment approach to the patient’s needs [49].

At the same time, the strengths of the present study are related to the same original real-world data, as they have been retrieved from a real hospital. Moreover, the analysis was conducted whilst taking into consideration three sample specialties (General Surgery, Orthopedics and Neurosurgery); however, it can be extended to all surgical specialties, resulting in additional benefits for the hospital.

## 9. Conclusions

The HTA study carried out recommends for the adoption of FAW technology to prevent inadvertent peri-operative hypothermia and to achieve significant benefits in patients undergoing surgery. In fact, the multi-dimensional analysis demonstrates the strategic relevance of the introduction of FAW in Italian clinical practice, its economic sustainability and feasibility, as well as the potentialities for process improvement, both from an economic and organizational perspective. It is hoped that, with the above-reported quantification of the benefits that can be obtained from the correct use of FAW technology, the process of adhering to guidelines will be further pursued.

## Figures and Tables

**Table 1:** Dimensions and features investigated in the qualitative analysis.

Dimensions	Features Investigated
Safety	(i) severe adverse events (ii) moderate adverse events (iii) general technology safety (iv) eventual safety risks to the healthcare professional (v) eventual environmental safety risks
Effectiveness	(i) impact of the device on maintaining temperature during surgery (ii) impact of the device on warming the patient during surgery (iii) impact of the device on reducing postoperative adverse events
Ethical Analysis	(i) accessibility of the technology (ii) accessibility of the technology to protected categories (iii) impact of the technology on waiting lists (iv) ability of the technology to generate health migration in case of use (v) existence of factors that could prevent a group or certain people from benefiting from the technology (vi) equity or inequity of the technology (vii) impact of accessibility to the patient by modification of hospitalization days (viii) impact of accessibility to the patient by modification of occurrence of complications (ix) impact of accessibility to the patient by modification of surgical time
Social Aspects	(i) ability of the technology to safeguard patient autonomy (ii) ability of the technology to safeguard human rights (iii) ability of the technology to safeguard human integrity (iv) ability of the technology to ensure human dignity (v) impact of the technology on social costs (vi) impact of the technology on improving the quality of life of patients (vii) impact of the technology on improving the quality of life of care-givers and family members (viii) impact of the technology on modifying adverse events for patients
Legal Aspects	(i) level of authorization (National/European/International) (ii) fulfillment of safety requirements (iii) infringement of intellectual property rights (iv) guarantees of production and use (v) subject to price control (vi) need to regulate the acquisition of the technology (vii) legislation covers its regulation for all categories of patients (viii) the user manual of the technology is comprehensive and technically complete (ix) the user manual of the technology is written in Italian
Short- and long-term qualitative Organizational Aspects	(i) use of the technology requires additional staff (during the intervention) (ii) use of the technology requires training of staff involved in the processes (iii) meetings required to manage the specific technology alternative (iv) time and learning curves (v) new spaces for the management of the technological alternatives (during and after the intervention) (vi) upgrade of the equipment for the usage of the technological alternatives (vii) purchase of consumables (viii) impact on internal processes within the relevant Operating Unit (ix) impact on the hospital’s purchasing processes (x) impact on the relations between Operating Units (xi) impact on PDT (Percorsi Diagnostico Terapeutici)/PDTA (Percorsi Diagnostico Terapeutici Assistenziali) processes (xii) life cycle impact of the technological alternative (xiii) ease of use of the technological alternative (xiv) ease of setting up the technological alternative in the pre-intervention phase (xv) ease of cleaning and sanitizing the technology alternative (xvi) impact of maintenance (corrective and scheduled) of the technology alternative (xvii) ease of availability of consumables for the technology alternative (xviii) impact of electricity consumption generated by use (xix) impact of heating quality of the technology alternative

**Table 2:** Validation of scientific evidence.

Article	Evaluation Scale	Results
Galvao, 2010 [32]	AMSTAR 2	Low Quality
Madrid, 2016 [17]	AMSTAR 2	High Quality
Shaw, 2017 [33]	AMSTAR 2	High Quality
Sumida, 2019 [34]	NEW CASTLE OTTAWA SCALE	High Quality
Engelen, 2011 [35]	JADAD	Medium Quality
Bezerra, 2020 [18]	JADAD	Medium Quality

**Table 3:** Safety and efficacy indicators.

	Hypothermia (Absence of Patient Warming Systems)	Normothermia (Appropriate Use of FAW Technology)	Referrals
**Safety Indicators**			
% of patients who develop surgical site infections	12.00%	3.00%	NICE [1]
% of patients who develop cardiac events	7.59%	3.45%	NICE [1]
% of patients who require transfusions	7.43%	6.24%	NICE [1]
**Efficacy Indicators**			
Average temperature at the end of surgery (°C—*p*-value < 0.001)	35.4 ± 0.1	36.7 ± 0.1	Frank, 1997 (from Madrid, 2016) [36]
Surgical site infections (% of patients who develop surgical site infections— *p*-value = 0.009)	19%	6%	Kurz, 1996 (from Madrid, 2016) [11]
Cardiovascular complications (% of patients who present cardiovascular complications— *p*-value = 0.02)	6.3%	1.4%	Frank, 1997 (from Madrid, 2016) [36]
Transfusions (amount of blood and blood components bags transfused during surgery and up to 48 h after— *p*-value < 0.05)	8	1	Schmied, 1996 (from Madrid, 2016) [37]
Intraoperative haemorrhage (blood ml—*p*-value = 0.043)	205 ± 209	146 ± 160	Pu, 2014 (from Madrid, 2016) [38]
Post-operative shivers (% of patients who report post-operative shivers— *p*-value = 0.034)	52.7%	32.7%	Pu, 2014 (from Madrid, 2016) [38]

**Table 4:** Economic evaluation—Activity Based Costing analysis and Cost-Effectiveness Analysis.

	Hypothermia	Normothermia (FAW)
	EUR	EUR
Pre-operative hospital stay	1058.03	1058.03
Surgery	1779.52	1779.52
FAW technology	0	7.13
Cost for adverse events: infections	331.99	83.00
Cost for adverse events: cardiac events	162.57	73.90
Cost for adverse events: transfusions	20.37	17.11
Intensive care hospital stay	5216.95	5216.95
Post-operative hospital stay	4813.72	3015.77
Total Costs	13,383.15	11,251.39
Surgical wound infection (% of patients who do not register infections)	81.0% (100–19%)	94.0% (100–6%)
Cardiovascular complications (% of patients who do not register cardiovascular complications)	93.7% (100%–6.3%)	98.6% (100–1.4%)
CEV (1)	16,522.53	11,969.70
CEV (2)	14,283.08	11.411–28

**Table 5:** Economic evaluation—Budget Impact Analysis (BIA).

Scenarios	Hypothemia	Normothermia	Total Cost of Hypothermic Path (EUR)	Total Cost of Normothermic Path (EUR)	Total Annual Cost (EUR)	Δ (EUR)	Δ%
AS-IS	97.57%	2.3%	111,567,868	2,336,032	113,903,900	-	-
TO-BE 1	50%	50%	57,173,244	48,066,493	105,239,737	−8,664,162	−7.61%
TO-BE 2	30%	70%	34,303,946	67,293,091	101,597,037	−12,306,863	−10.80%
TO-BE 3	15%	85%	17,151,973	81,713,039	98,865,012	−15,038,888	−13.20%

**Table 6:** Impact on reduction in hospitalization days.

Scenarios	Hypothemia	Normothermia	Days of Hospitalization Hypothermia	Days of Hospitalization Normothermia	Total Days of Hospitalization	Δ (gg)	Δ%
AS-IS	97.57%	2.43%	70,442	1215	71,657	-	-
TO-BE 1	50%	50%	36,098	24,991	61,090	−10,567	−14.75%
TO-BE 2	30%	70%	21,659	34,988	56,647	−15,010	−20.95%
TO-BE 3	15%	85%	10,830	42,485	53,315	−18,342	−25.60%

**Table 7:** Results of qualitative analysis.

Dimensions	Features Investigated	Hypothermia	Normothermia (FAW)
*Safety*	(i) severe adverse events	−0.10	1.50
	(ii) moderate adverse events	−0.30	1.40
	(iii) general technology safety	−0.20	1.90
	(iv) eventual safety risks to the healthcare professional	−1.00	1.00
	(v) eventual environmental safety risks	−0.70	1.10
	average value for the safety dimension	−0.46	1.38
*Effectiveness*	(i) impact of the device on maintaining temperature during surgery	−1.5	1.70
	(ii) impact of the device on warming the patient during surgery	−2.10	1.70
	(iii) impact of the device on reducing postoperative adverse events	0.00	1.80
	average value for the effectiveness Dimension	−1.20	1.73
*Ethical Analysis*	(i) accessibility of the technology	−0.10	1.50
	(ii) accessibility of the technology to protected categories	−0.70	1.40
	(iii) impact of the technology on waiting lists	−0.10	0.50
	(iv) ability of the technology to generate health migration in case of use	−0.40	0.50
	(v) existence of factors that could prevent a group or certain people from benefiting from the technology	−0.10	0.10
	(vi) equity or inequity of the technology	−0.10	1.60
	(vii) impact of accessibility to the patient by modification of hospitalization days	−0.60	1.50
	(viii) impact of accessibility to the patient by modification of occurrence of complications	−0.60	1.50
	(ix) impact of accessibility to the patient by modification of surgical time	−0.60	0.70
	average value for the ethical dimension	–0.37	1.03
*Social Aspects*	(i) ability of the technology to safeguard patient autonomy	−0.60	1.50
	(ii) ability of the technology to safeguard human rights	−0.70	0.70
	(iii) ability of the technology to safeguard human integrity	−0.90	1.70
	(iv) ability of the technology to ensure human dignity	−0.80	1.60
	(v) impact of the technology on social costs	−0.50	0.70
	(vi) impact of the technology on improving the quality of life of patients	−0.80	1.80
	(vii) impact of the technology on improving the quality of life of care-givers and family members	−0.10	0.80
	(viii) impact of the technology on modifying adverse events for patients	−0.80	1.70
	average value for the social dimension	−0.65	1.31
*Legal Aspects*	(i) level of authorization (National/European/International)	−0.60	1.50
	(ii) fulfillment of safety requirements	−0.30	1.90
	(iii) infringement of intellectual property rights	0.00	0.00
	(iv) guarantees of production and use	−0.40	1.40
	(v) subject to price control	0.10	0.70
	(vi) need to regulate the acquisition of the technology	0.10	1.10
	(vii) legislation covers its regulation for all categories of patients	−0.10	1.50
	(viii) the user manual of the technology is comprehensive and technically complete	−0.10	1.70
	(ix) the user manual of the technology is written in Italian	−0.10	1.50
	average value for the legal dimension	−0.16	1.26
*Short-term qualitative Organizational Aspects*	(i) use of the technology requires additional staff (during the intervention)	0.10	−0.40
	(ii) use of the technology requires training of staff involved in the processes	−0.10	0.90
	(iii) meetings required to manage the specific technology alternative	0.40	0.40
	(iv) time and learning curves	0.00	1.60
	(v) new spaces for the management of the technological alternatives (during and after the intervention)	−0.10	1.00
	(vi) upgrade of the equipment for the usage of the technological alternatives	−0.60	0.40
	(vii) purchase of consumables	−0.20	0.20
	(viii) impact on internal processes within the relevant Operating Unit	−0.10	0.60
	(ix) impact on the hospital’s purchasing processes	0.20	0.30
	(x) impact on the relations between Operating Units	−0.10	0.20
	(xi) impact on PDT (Percorsi Diagnostico Terapeutici)/PDTA (Percorsi Diagno-stico Terapeutici Assistenziali)	−0.30	0.40
	(xii) life cycle impact of the technological alternative	−0.10	1.20
	(xiii) ease of use of the technological alternative	−0.20	1.60
	(xiv) ease of setting up the technological alternative in the pre-intervention phase	−0.30	0.90
	(xv) ease of cleaning and sanitizing the technology alternative	−0.20	0.90
	(xvi) impact of maintenance (corrective and scheduled) of the technology alternative	−0.20	0.80
	(xvii) ease of availability of consumables for the technology alternative	−0.10	0.70
	(xviii) impact of electricity consumption generated by use	−0.20	−0.30
	(xix) (xix) impact of heating quality of the technology alternative	−0.30	1.40
	average value for the organizational dimension (short t.)	−0.17	0.67
*Long-term qualitative Organizational Aspects*	(i) use of the technology requires additional staff (during the intervention)	−0.20	−0.20
	(ii) use of the technology requires training of staff involved in the processes	−0.40	0.40
	(iii) meetings required to manage the specific technology alternative	−0.30	0.50
	(iv) time and learning curves	−0.30	1.20
	(v) new spaces for the management of the technological alternatives (during and after the intervention)	−0.20	0.60
	(vi) upgrade of the equipment for the usage of the technological alternatives	−0.20	0.80
	(vii) purchase of consumables	−0.20	0.50
	(viii) impact on internal processes within the relevant Operating Unit	−0.20	1.20
	(ix) impact on the hospital’s purchasing processes	−0.20	0.30
	(x) impact on the relations between Operating Units	−0.10	0.50
	(xi) impact on PDT (Percorsi Diagnostico Terapeutici)/PDTA (Per-corsi Diagno-stico Tera-peutici Assistenziali)	−0.40	0.70
	(xii) life cycle impact of the technological alternative	−0.20	0.80
	(xiii) ease of use of the technological alternative	−0.20	1.40
	(xiv) ease of setting up the technological alternative in the pre-intervention phase	−0.20	1.60
	(xv) ease of cleaning and sanitizing the technology alternative	−0.10	1.00
	(xvi) impact of maintenance (corrective and scheduled) of the technology alternative	−0.20	0.90
	(xvii) ease of availability of consumables for the technology alternative	−0.20	0.80
	(xviii) impact of electricity consumption generated by use	−0.30	0.30
	(xix) impact of heating quality of the technology alternative	−0.20	1.80
	average value for the organizational dimension (long t.)	−0.23	0.79

## Data Availability

Sources of data used for analysis are cited in the bibliography. In addition, anonymized and aggregated data drawn from an Italian reality were used, in order to perform the analyses necessary to support the study.The data that support the findings of this study are available from the corresponding author, [G.Z.], upon reasonable request.
